# Googling the Guggul (Commiphora and Boswellia) for Prevention of Chronic Diseases

**DOI:** 10.3389/fphar.2018.00686

**Published:** 2018-08-06

**Authors:** Ajaikumar B. Kunnumakkara, Kishore Banik, Devivasha Bordoloi, Choudhary Harsha, Bethsebie L. Sailo, Ganesan Padmavathi, Nand K. Roy, Subash C. Gupta, Bharat B. Aggarwal

**Affiliations:** ^1^Cancer Biology Laboratory, DBT-AIST International Laboratory for Advanced Biomedicine (DAILAB), Department of Biosciences and Bioengineering, Indian Institute of Technology Guwahati, Assam, India; ^2^Department of Biochemistry, Institute of Science, Banaras Hindu University, Varanasi, India; ^3^Inflammation Research Center, San Diego, CA, United States

**Keywords:** guggul, guggulsterone, *boswellia*, boswellic acid, cancer, *commiphora*, chronic diseases

## Abstract

Extensive research during last 2 decades has revealed that most drugs discovered today, although costs billions of dollars for discovery, and yet they are highly ineffective in their clinical response. For instance, the European Medicines Agency has approved 68 anti-cancer drugs, and out of which 39 has reached the market level with no indication of increased survival nor betterment of quality of life. Even when drugs did improve survival rate compared to available treatment strategies, most of these were found to be clinically insignificant. This is a fundamental problem with modern drug discovery which is based on thinking that most chronic diseases are caused by alteration of a single gene and thus most therapies are single gene-targeted therapies. However, extensive research has revealed that most chronic diseases are caused by multiple gene products. Although most drugs designed by man are mono-targeted therapies, however, those designed by “mother nature” and have been used for thousands of years, are “multi-targeted” therapies. In this review, we examine two agents that have been around for thousands of years, namely “guggul” from *Commiphora* and *Boswellia*. Although we are all familiar with the search engine “google,” this is another type of “guggul” that has been used for centuries and being explored for its various biological activities. The current review summarizes the traditional uses, chemistry, *in vitro* and *in vivo* biological activities, molecular targets, and clinical trials performed with these agents.

## Introduction

**Figure 1:**
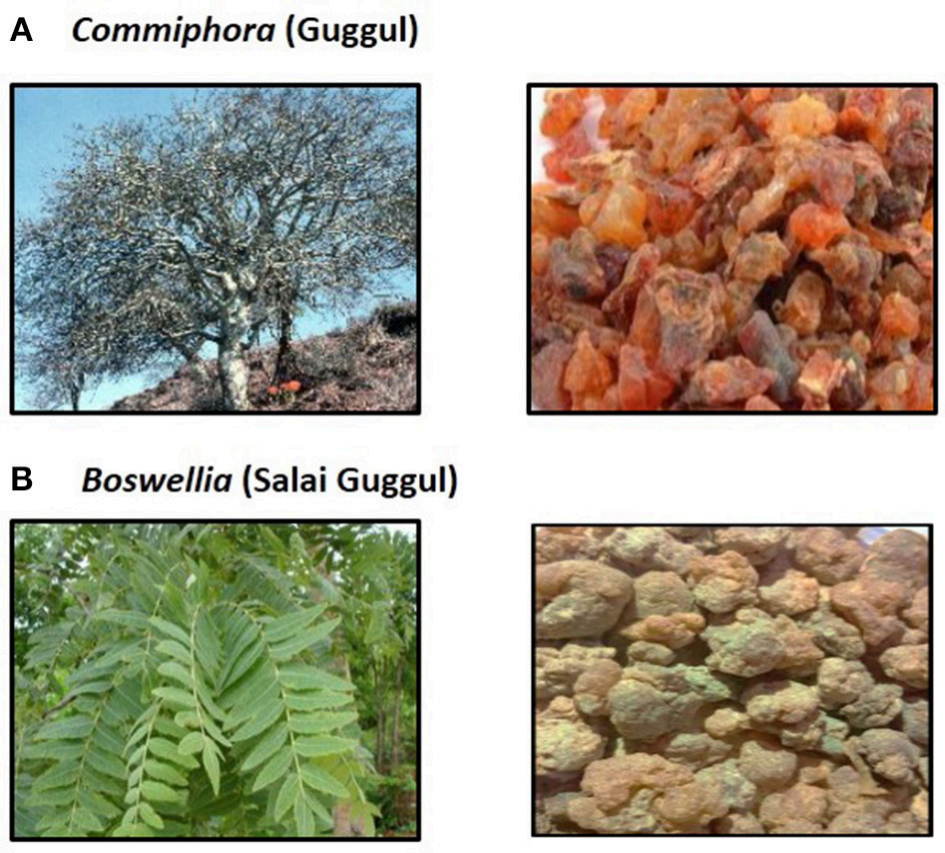
**(A)** Commiphora (Mark W. Skinner/www.discoverlife.org) and Gum guggul (http://www.varionlife.com). **(B)** *Boswellia* (Pankaj Oudhia/www.discoverlife.org) and Salai guggul.

Despite the remarkable advances made in the field of therapies for chronic diseases including cancer over the last few decades, they still present a major health burden and are the prime cause of death across the world. Most of the chronic illnesses are caused by the deregulation of multiple genes; however majority of the drugs approved by Food and Drug Administration (FDA) target single gene product or pathway only. This displays one of the major drawbacks of these synthetic drugs. In addition, these drugs are associated with different adverse side effects and hence not tolerable by patients (Siddiqui et al., 1984; Sarup et al., 2015; Kunnumakkara et al., 2017; Banik et al., 2018). Therefore, there is an urgent need to identify novel, safe, and multi-targeted agents for the prevention and treatment of these diseases (Bordoloi et al., 2016; Kunnumakkara et al., 2018). It has been well-evidenced that natural products are effective, multi-targeted, and extremely safe as they are the roots of many traditional systems of medicine such as Ayurveda, Unani, Siddha, traditional Chinese medicine etc. (Shishodia et al., 2008; Harsha et al., 2017). One such medicine of enormous use in Ayurveda is “*Guggul*.” Guggul is the gum resin obtained from two different plants *Commiphora* and *Boswellia*, produced by drying the white sap of 15–20 years old tree for a year (Figure 1; Hanus et al., 2005).

The history of guggul goes as far back as 1700 BC. Ancient script on medicine and surgery; *Sushrut Samhita*, describes that guggul when taken orally can cure internal tumors, malignant sores, obesity, liver dysfunction, intestinal worms, leucoderma, sinus, and edema. It is also used as an Ayurvedic medicine for the prevention and treatment of various other diseases such as inflammatory bowel disease (IBD), ulcers, arthritis, cardiovascular diseases (CVDs), diabetes etc. (Shishodia et al., 2008). The main ingredients of guggul are guggulsterone (GS) and boswellic acid (BA) which are obtained from *Commiphora* and *Boswellia* respectively. It also contains a huge number of lignans and ketosterols, which contributes to the vivid health beneficiary effects of guggul (Arora et al., 1971, 1972; Kimura et al., 2001; Zhu et al., 2001; Francis et al., 2004).

According to Pubmed; “google,” there are 449 publications on *Commiphora*, 519 on *Boswellia*, 207 on guggulsterone, 329 on boswellic acid, and 90 on guggul with earliest being in 1960 describing the “Antiarthritic and anti-inflammatory activity of the gum “guggul”; and in 1969 on “Analgesic effect of the gum resin from *Boswellia serata*.” Some of the major species include *Commiphora wightii* (guggul), *Commiphora mukul, Commiphora gileadensis, Boswellia serrata* (salai guggul), *Boswellia carterii, Boswellia sacra* (source of frankincense & gum resin), *Boswellia ovalifoliolata, Boswellia dalzielii, Boswellia frerean*, and *Boswellia thurifera*. What is common among all these plants and their products is that all of them exhibit anti-inflammatory activities, although to a variable extent. The current review describes the traditional uses, chemistry, molecular targets, *in vitro, in vivo* and clinical studies of guggul isolated from *Commiphora* and *Boswellia*.

## Source and chemical constituents of *commiphora* and *boswellia*

**Figure 2:**
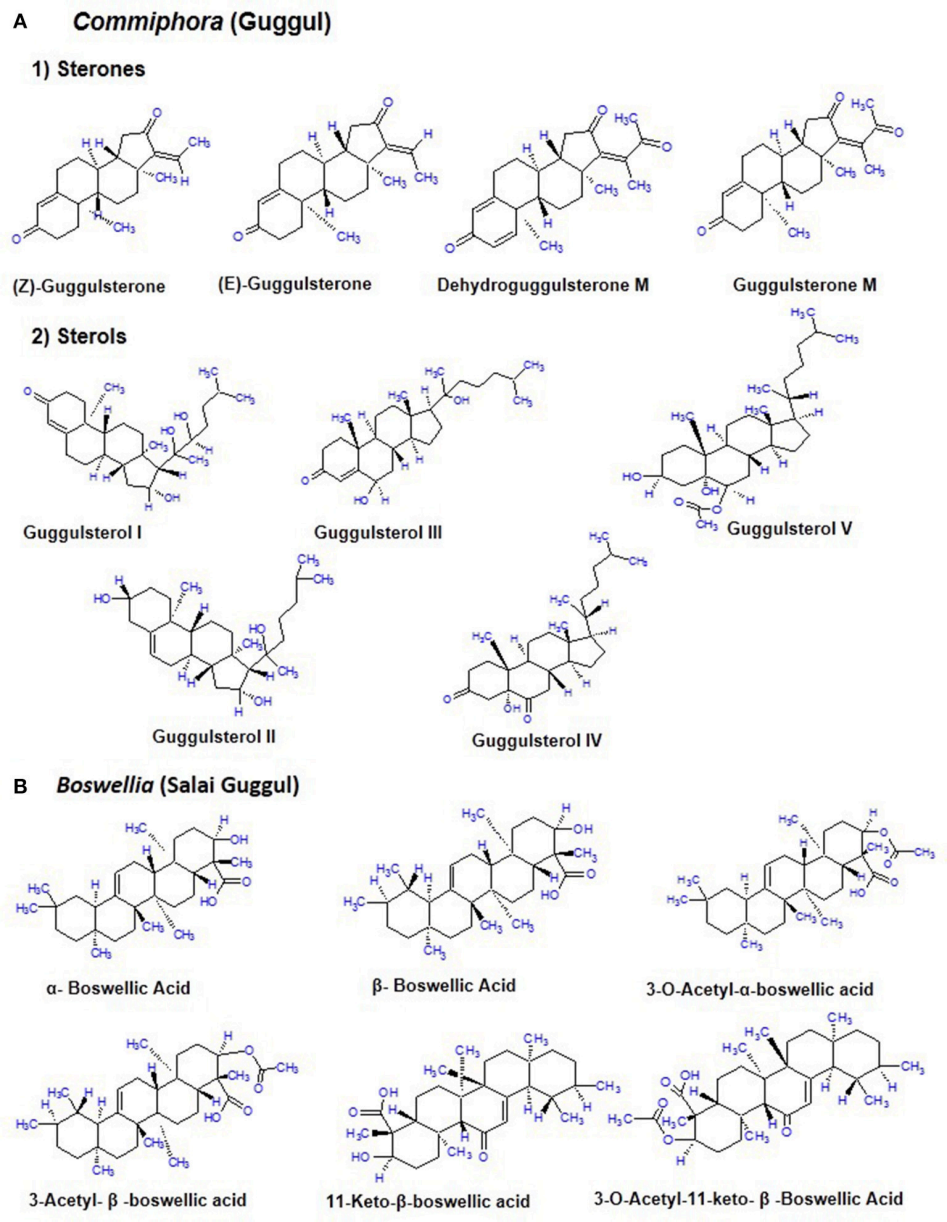
Chemical constituents of *Commiphora* (guggul) and *Boswellia* (Salai guggul) **(A)** *Commiphora* **(B)** *Boswellia*.

The guggul tree which belongs to the family *Burseraceae*, is mainly found in the dry regions of the Indian subcontinent mainly India, Pakistan and Bangladesh. The oleogum resin of *C. mukul* (guggul tree) is a yellowish substance that is tapped during winter and ~700–900 g of resin is obtained from each tree (Deng, 2007; Shishodia et al., 2015; Yamada and Sugimoto, 2016). The guggul or balsam or the oleo gum resin is found in the balsam canals in the phloem of the large veins of leaf and base of the stem. It is a complicated mixture of minerals, gum, terpenes, sterols (Guggulsterol -I,-II,-III,-IV,-V), essential oils, sterones (Z-, E-, M-guggulsterone, and dehydroguggulsterone-M), ferrulates, lignans, and flavanones. The ethyl acetate soluble fraction also known as guggulipid, consists of various bioactive components like diterpenoids, triterpenoids, steroids, lignans, and fatty tetrol esters. Based on the pH gradient, further fractionation yields 95% neutral, 4% acidic, and 1% basic fractions. The neutral fraction when subjected to further fractionation produces 88% non-ketonic and 12% ketonic fractions. A large number of steroids including the two isomers E-(cis-) and Z-(trans-) GS [4, 17(20)-pregnadiene-3, 16-dione] were obtained from the ketonic fraction. Nearly 5% guggulipid and 2% gum guggul by weight is present in the GS (Figure 2A; Deng, 2007; Shishodia et al., 2008, 2015; Sarup et al., 2015).

Phenolics are common natural products found in plants and possess substantial antioxidant and anti-inflammatory effects. Various phenolic compounds such as hydroxybenzoic acid derivatives such as gallic acid, protocatechuic acid, gentisic acid, vanillic acid, p-hydroxy benzoic acid, syringic acid, ellagic acid, and cinnamic acid derivatives which include caffeic acid, chlorogenic acid, ferulic acid, sinapic acid (SA), and p-coumaric acid are largely present in plants. These phenolic compounds are predominantly available in guggul as well, which in part contributes to its immense biological function against diverse human chronic diseases (Hazra et al., 2018).

Guggulsterone is the only known antagonist of farnesoid X receptor (FXR). This FXR, also known as NR1H4 (nuclear receptor subfamily 1, group H, member 4), is a bile acid receptor (BAR). Bioinformatics studies (molecular docking simulation) revealed that GS binds to FXR and nuclear factor-kappa B (NF-κB) and it docks into two non-canonical binding sites of FXR, helix 1-loop-helix 2 loop and parts of helix—helix 8 including helix 8-loop-helix 9 (Meyer et al., 2005; Yang et al., 2014). Different bile acids and chenodeoxycholic acids act as natural ligand for FXR, whose expression is elevated in the liver and intestine. When FXR binds to its ligand, it gets activated and reaches the cell nucleus, where it forms a heterodimer with RXR. This heterodimer binds to the hormone response elements on DNA and regulates various genes. FXR activation downregulates cholesterol 7 alpha-hydroxylase (CYP7A1), the rate-limiting enzyme in bile acid synthesis from cholesterol by inducing the expression of small heterodimer partner (SHP) which in turn inhibits the transcription of CYP7A1 gene. While obeticholic acid, fexaramine, cafestol, and chenodeoxycholic acid act as agonist of FXR; GS, from the gum resin of guggul has also been confirmed to inhibit pro-inflammatory signals, together with transcription factor NF-κB (Sharma and Sharma, 1977; Urizar et al., 2002; Shishodia et al., 2008; Yamada and Sugimoto, 2016). Another study reported that the inhibitory activity of NF-κB is due to the binding of GS to the RH domain of NF-κB precursor protein p105 containing important sequences for DNA binding and dimerization (Khan et al., 2013).

*B. serrata*, commonly known as salai guggul, Indian olibanum, loban, or kundur, belongs to the *Burseraceae* family and is found in dry mountainous regions of India, Northern Africa, and the Middle East. *Burseraceae* family includes 17 genera and 600 species of plants. The genus *Boswellia* has 25 different species distributed throughout the tropical regions. *B. serrata* is one such medicinal plant which exhibits immense potential to combat various chronic disorders. The active pharmacological principle of the oleo gum resin from the trees of different *Boswellia* species is the BA (Büchele et al., 2003; Du et al., 2015; Roy et al., 2016). The gum resin of the *Boswellia* species mainly consists of mucus, resin acids, and volatile oil with different quantitative composition from species to species. The gum resin of salai guggul contains pentacyclic triterpenic acids, namely α-boswellic acids, β-boswellic acids, γ-boswellic acid, acetyl-β-boswellic acid, 11-keto-β-boswellic acid (KBA), acetyl-11-keto-β-boswellic acid (AKBA), and tetracyclic triterpenic acids like tirucallic acids viz 3-oxotirucallic acid, 3-hydroxytirucallic acid, and 3-acetoxytirucallic acid (Figure 2B). Other oleo gum resin compounds which display biological activities are: betulinic acid, lupenoic acid, epi-lupeol, isoincensole, isoincensole acetate and 1-ursene-2-diketone-incensole acetate along with few other terpenes that can be found in volatile oil (Du et al., 2015; Ammon, 2016; Roy et al., 2016).

## Molecular targets of *commiphora* and *boswellia*

**Figure 3:**
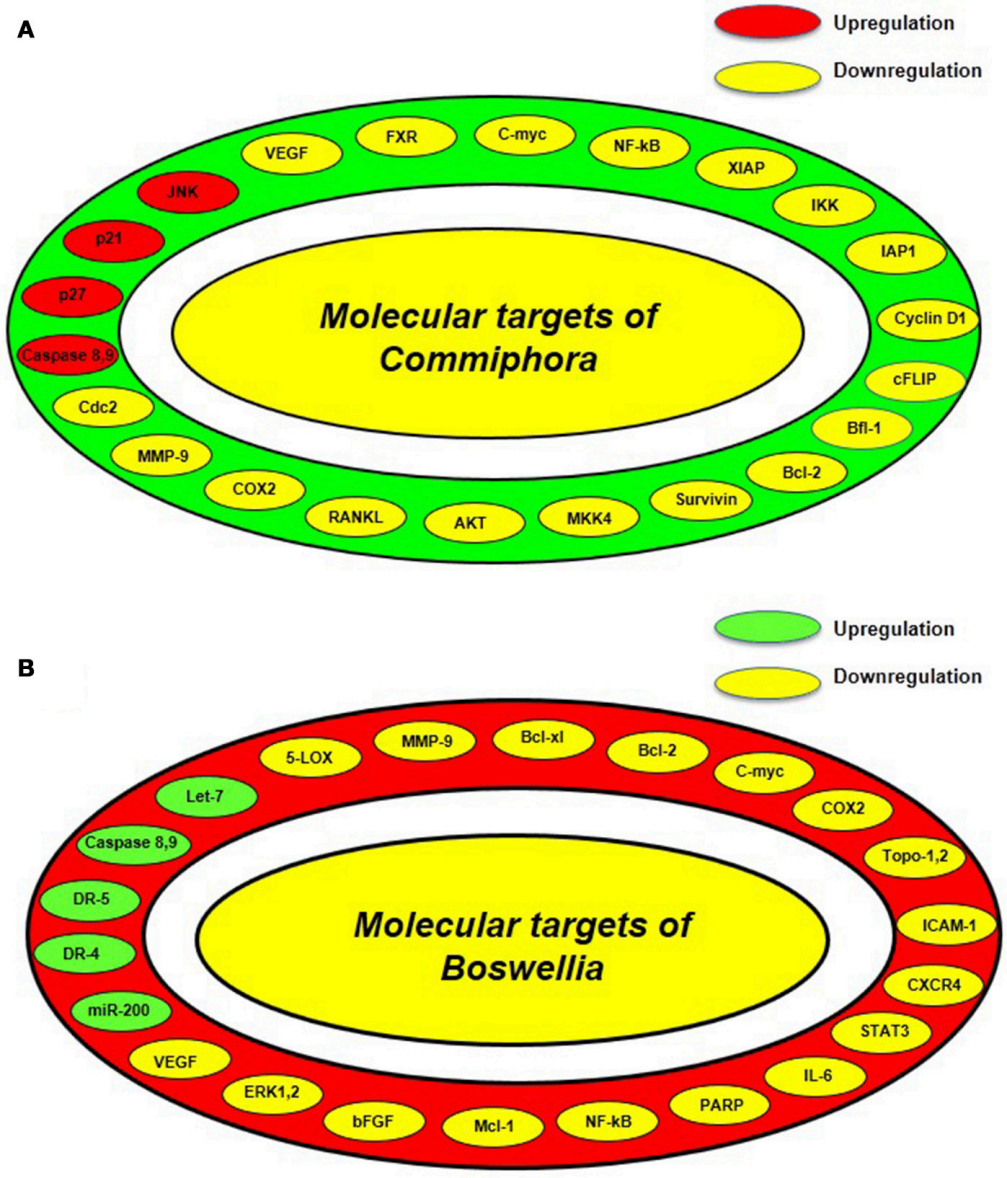
Various molecular targets of guggul from *Commiphora* and *Boswellia* **(A)** Molecular targets of guggul from *Commiphora* includes Bcl2, B-cell lymphoma 2; CDC 2, cell division cycle kinase 2; c-FLIP, cellular caspase-8 (FLICE)-like inhibitory protein; COX, cyclooxygenase; FXR, farnesoid X receptor; IKK, IκB kinase; IAP, Inhibitors of apoptosis *proteins*; JNK, c-Jun N-terminal kinase; MKK4, mitogen-activated protein kinase kinase 4; MMP, matrix metalloproteinase; NF-κB, nuclear factor-κB; RANKL, Receptor activator of nuclear factor kappa-B ligand; VEGF, vascular endothelial growth factor; XIAP, x-linked inhibitor of apoptosis protein. **(B)** Molecular targets of guggul from *Boswellia* includes Bcl-2, B-cell lymphoma 2; Bcl-xL, B-cell lymphoma-extra-large; CXCR-4, C-X-C chemokine receptor type 4; DR, Death receptor; 5-LOX, 5-Lipoxygenase; MMP, Matrix metalloproteinase; NF-κB, nuclear factor-κB; Topo, Topoisomerase; ICAM-1, Intercellular adhesion molecule 1; STAT-3, Signal transducer and activator of transcription 3; IL-6, Interleukin 6; PARP, Poly ADP ribose polymerase; Mcl-1, Myeloid leukemia cell differentiation protein; bFGF, Basic fibroblast growth factor; ERK-1,-2, Extracellular signal-regulated kinases.

GS suppresses the physiological action of the FXR which is a nuclear hormone receptor that controls the synthesis and transport of bile acid (Sinal and Gonzalez, 2002; Urizar et al., 2002). However, it increases the transcription of bile salt export pump (BSEP) which is majorly involved in hepatic bile acid transport (Cui et al., 2003). Besides regulating transport of bile acid, GS is a potent anti-inflammatory agent which suppresses LPS-induced NO production (Meselhy, 2003). GS has also been reported to inhibit the activation of NF-κB by suppressing the levels of receptor activator of NF-κB ligand (RANKL) (Ichikawa and Aggarwal, 2006). In 2004, Shishodia and group reported that GS suppressed the activation of NF-κB and IκB-α kinase and exhibited antiproliferative activity by inhibiting c-Myc and cyclin D1. Furthermore, GS has also been found to exert anti-metastatic effect through reducing the levels of MMP-9, COX-2, and VEGF (Shishodia and Aggarwal, 2004). This group also reported that GS induced apoptosis by modulating the expression of anti-apoptotic genes, IAP1, XIAP, Bcl-2, cFLIP, Bfl-1/A1, and survivin (Shishodia and Aggarwal, 2004). Further, GS has also been found to induce tumor cell apoptosis by activating the apoptotic genes, caspase-3,−8,−9, and inducing the release of cytochrome c, cleavage of bid and PARP. This was controlled by activated mitogen-activated protein kinase 4 (MKK4) mediated upregulation of c-Jun N-terminal kinase (JNK) and suppression of Akt. The antiproliferative activity of GS was found to be supported by reduced levels of cyclin D1, cdc2, and simultaneous upregulation of cyclin-dependent kinase inhibitors p21 and p27 (Figure 3A; Shishodia et al., 2007).

Boswellic acid is known to inhibit leukotriene synthesis by inhibiting 5-lipoxygenase (5-LOX) (Safayhi et al., 1992, 1995; Ammon et al., 1993). This 5-LOX inhibitor has also been found to reduce the activity of human leukocyte elastase (HLE) *in vitro* (Safayhi et al., 1997). Suppression of these molecules contributes to anti-inflammatory action of *Boswellia*. *Boswellia* is also known to induce apoptosis in cancer cells. In 2007, Bhushan and group reported that a triterpenediol from *B. serrata* induced apoptosis in HL-60 cells through both intrinsic and extrinsic pathways (Bhushan et al., 2007). In the first case, the triterpenediol was found to disturb the mitochondrial membrane potential, reduce Bcl-2/Bax ratio and cause release of AIF, Smac/DIABLO, and cytochrome c from the mitochondria along with suppression of survivin and upregulation of caspases-3,−8, and−9, thereby leading to the cleavage of ICAD and PARP while in the second case, the oxidative stress generated in the cells due to excessive ROS and NO production triggered the activation of TNF-R1 and DR4 followed by activation of caspase-8. Another study in multiple myeloma cells also suggested that BA acetate induces apoptosis by upregulating death receptor proteins, DR4 and DR5 which subsequently leads to the activation of caspase-8 followed by caspase-3 (Xia et al., 2005). The role of DR5-mediated pathway which involves activation of CAAT/enhancer binding protein homologous protein (CHOP) was reported in AKBA-mediated apoptosis of prostate cancer cells (Lu et al., 2008). Caspase-8 activation has also been reported in other BA-induced apoptosis studies (Liu et al., 2002a,b). In 2002, Park et al. hypothesized that AKBA contributed in the process of proliferation and apoptosis of tumors by inhibiting platelet-derived growth factor (PDGF)-stimulated extracellular signal-regulated kinase 1 and 2 (ERK-1 and ERK-2) (Park et al., 2002b). BA mediated apoptosis has also been evident in cancer cells via activation of p21, an important cell cycle regulator protein (Glaser et al., 1999; Liu et al., 2006). Apart from this, AKBA has been found to interfere with IL-6-induced STAT3 signaling via protein tyrosine phosphatase SHP-1 subsequently causing downregulation of cyclin D1, Bcl-2, Bcl-xL, Mcl-1, and VEGF, thus impeding proliferation, survival and angiogenesis of multiple myeloma cells (Kunnumakkara et al., 2009). Moreover, BA has also been found to suppress metastatic growth factor, basic fibroblast growth factor (bFGF), chemokine receptor; CXCR4 and angiogenic factor; VEGFR 2 (Singh et al., 2007; Pang et al., 2009; Park et al., 2011a). Further, *in vivo* studies have unveiled that BA regulates proliferation and metastasis of cancer cells by downregulating other targets like COX-2, c-Myc, cyclin D1, MMP-9, VEGF, ICAM-1, Bcl-2, Bcl-xL, survivin, and cellular inhibitor of apoptosis protein 1 (IAP-1) (Park et al., 2011a,b; Yadav et al., 2012). Most of these genes are regulated by the transcription factor, NF-κB which is also downregulated by BA (Syrovets et al., 2005a,b; Takada et al., 2006). Furthermore, BA has also been shown to regulate the activity of P-glycoprotein (Pgp) which is an important class of drug transporters (Weber et al., 2006). It is also an inhibitor of topoisomerases I and II in cancer cells (Hoernlein et al., 1999; Syrovets et al., 2000; Zhao et al., 2003). The anticancer activity of this potential compound also involves regulation of let-7 and miR-200 microRNA family (Figure 3B; Takahashi et al., 2012).

## Therapeutic properties of guggul

**Figure 4:**
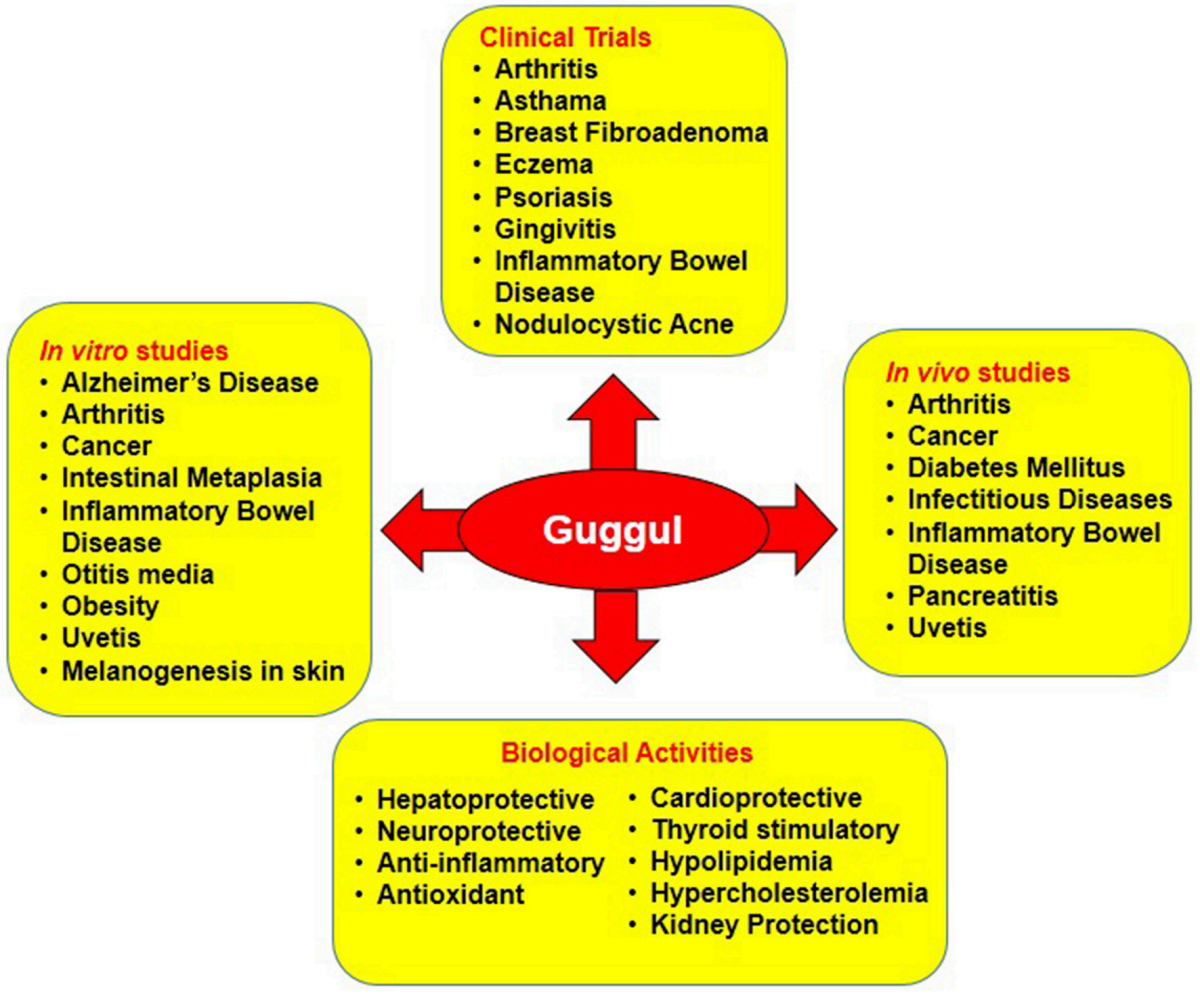
*In vitro, in vivo* and clinical studies on the biological activities of guggul against diverse chronic diseases.

Congregate evidences show guggul to be profoundly effective against diverse chronic diseases such as Alzheimer's disease, arthritis, cancer, pancreatitis, IBD, dermatitis, diabetes, infectious diseases, intestinal metaplasia, otitis media, respiratory diseases, asthma, psoriasis, gingivitis etc. Besides, it also exerts hepatoprotective, neuroprotective, anti-inflammatory, anti-oxidant, cardioprotective, hypolipidemia, and thyroid stimulatory effect by targeting multiple signaling pathways (Table 1; Figure 4).

**Table 1:** *In vitro* biological activities of guggul (*Commiphora* and *Boswellia*) against various chronic diseases.

**Disease**	**Mechanism of action**	**References**
Arthritis	↓RANKL-induced NF-κB activation	Ichikawa and Aggarwal, 2006^c^
	↓IRF3	Youn et al., 2009^c^
	↓NF-κB	Zhang et al., 2016^c^
Barrett's esophagus	↓FXR	De Gottardi et al., 2006^c^
**CANCER**		
Bladder cancer	↑EGR1, ↑ATF3, ↑DDIT3	Frank et al., 2009^d^
Brain cancer	↑p21	Glaser et al., 1999^d^
	↓Erk-1, ↓Erk-2	Park et al., 2002a,b^d^
	–	Hostanska et al., 2002^d^
Breast cancer	↓MDR	Xu et al., 2011^c^
	↓MDR	Xu et al., 2012^c^
	↓NF-κB; ↓IGF1-Rβ; ↓ERα	Choudhuri et al., 2011^c^
	↓Wnt/β-Catenin; ↓cyclin D1; ↓C-myc	Jiang et al., 2013^c^
	↑Ho-1; ↑Nrf-2; ↑ROS & ↑p-Akt	Almazari et al., 2012^c^
	↑BCRP; ↓MDR	Kong et al., 2015^c^
Cervical cancer	↑P-gp & MRP1	Nabekura et al., 2008^c^
	↓PARP; ↓NF-κB	Qurishi et al., 2012^d^
Cholangiocarcinoma	↓Survivin; ↓Bcl-2	Zhong et al., 2015^c^
	↓ROS/JNK	Zhong et al., 2016^c^
Colorectal cancer	↓STAT3 & VEGF	Kim et al., 2008^c^
	↓NF-κB; ↓IGF1-Rβ; ↓ERα	Choudhuri et al., 2011^c^
	↑Caspase-8	Liu et al., 2002b^d^
	↑p21; ↓cyclin D1,-E;↓CDK-2,- 4	Liu et al., 2006^d^
	↓PARP	Qurishi et al., 2012^d^
	↑let-7 and miR-200 families	Takahashi et al., 2012^d^
	↑SAMD14; ↑ SMPD3, ↓DNMT activity	Shen et al., 2012^d^
Esophageal cancer	↓CdX2	Yamada et al., 2014^c^
	↓NF-κB; ↓COX-2	Yamada et al., 2014^c^
Gall bladder cancer	↓NF-κB	Yang et al., 2012^c^
Glioma	↓Ras; ↓NFκB	Dixit et al., 2013^c^
	↓Topoisomerase I	Hoernlein et al., 1999^d^
Head and Neck	↑JNK; ↓Akt	Shishodia et al., 2007^c^
	↓STAT3	Li et al., 2009^c^
	↓Bcl-2; ↓XIAP; ↓cyclin D1; ↓c-myc	Macha et al., 2010^c^
	↓PI3K/Akt	Macha et al., 2011b^c^
	↓NF-κB; ↓STAT3	Macha et al., 2011a^c^
	↓p-STAT3; ↓STAT3	Leeman-Neill et al., 2009^c^
Leukemia	↑JNK; ↓Akt	Shishodia et al., 2007^c^
	↓ NF-κB ↓ IKK	Shishodia and Aggarwal, 2004^c^
	↓BAR	Wu et al., 2002^c^
	↑Externalization of PS	Samudio et al., 2005^c^
	↓ P-gp	Xu et al., 2012^c^
	–	Jing et al., 1999^d^
	–	Hostanska et al., 2002^d^
	↑Caspase-8, ↑DR4, ↑DR5	Xia et al., 2005^d^
	↓NF-κB	Takada et al., 2006^d^
	↓Topoisomerase I, ↓Topoisomerase II	Chashoo et al., 2011^d^
	↓PI3K/Akt/Hsp-90 cascade	Khan et al., 2012^d^
	-	Shao et al., 1998
	-	Huang et al., 2000^c^
	↓P-gp; ↓COX-2; ↓Prostaglandin E2	Xu et al., 2014a
Liver cancer	↓Cox-2; ↓P-gp	Xu et al., 2017^c^
	↑CHOP-dependent DR5	Moon et al., 2011^c^
	↓TGF-β1; ↓VEGF	Shi et al., 2015^c^
	↓BAR	Wu et al., 2002^c^
	↓CYP7A1	Owsley and Chiang, 2003^c^
	↑Caspase-8	Liu et al., 2002a^d^
Lung cancer	↑JNK; ↓Akt	Shishodia et al., 2007^c^
	↓PARP	Qurishi et al., 2012^d^
Melanoma	↓Tyrosinase	Koo et al., 2012^c^
	↑JNK; ↓Akt	Shishodia et al., 2007^c^
	↓Topoisomerase II, ↓MMPs	Zhao et al., 2003^d^
Meningioma	↓Erk-1, ↓Erk-2	Park et al., 2002a^d^
Myeloma	↓STAT3	Kunnumakkara et al., 2009^d^
	↓p-STAT3; ↓pJAK2; ↓p-c-Src; ↓SHP-1;	Ahn and Youn, 2008^c^
	↓STAT3; ↓Bcl-2; ↓Mcl-1; ↓cyclin D1;	
	↓VEGF; ↑Caspase-3 and ↓PARP	
Pancreatic cancer	↓NF-κB; ↓IGF1-Rβ; ↓ERα	Choudhuri et al., 2011^c^
	↓NF-κB	Park et al., 2011b^d^
Prostate cancer	↑JNK	Xiao et al., 2011^c^
	↓JAK/STAT	Macha et al., 2013^c^
	↑Bax; ↑Bak	Singh et al., 2005^c^
	↑PSA	Burris et al., 2005^c^
	↑JNK	Singh et al., 2007b^c^
	↓NF-κB	Syrovets et al., 2005b^d^
	↑Caspase 3	Büchele et al., 2006^d^
	↑DR5, ↑CHOP, ↑caspase-8, ↓PARP	Lu et al., 2008^d^
	↓AR, ↑p21, ↓cyclin D1	Yuan et al., 2008^d^
	↓mTOR	Morad et al., 2013^d^
	↓MMP, ↓PARP-1	Pathania et al., 2015^d^
	↓VEGF, ↓FGF, ↓G-CSF, ↓MMP-2	Xiao and Singh, 2008^c^
	↓IL-17, ↓VEGF-R2	
Neuroblastoma	↓PARP	Qurishi et al., 2012^d^
Cardiotoxicity	↓Caspase-3	Wang et al., 2012^c^
Chikungunya	↓Entry of CHIKV Env pseudotyped lentiviral vectors	von Rhein et al., 2016^d^
Gastric intestinal metaplasia	↓CdX2	Xu et al., 2010^c^
Hepatic fibrosis	↓NF-κB	Kim et al., 2013^c^
Kidney injury in systemic infection	↓NF-κB	Kim et al., 2016^c^
Nephrotoxicity	↓MAPK	Lee et al., 2017^c^
Neuroinflammation	↓IκBα - ↓NF-κB	Huang et al., 2016^c^
Obesity	↑Caspase-3; ↓PPARγ2, ↓C/EBP-α,-β	Yang et al., 2008^c^
Otitis media	↓NF-κB	Song et al., 2010^c^

*AR, Androgen receptor; ATF3, Activating transcription factor 3; BAR, Bile acid receptor; BCRP, Breast cancer resistance protein; c, Commiphora; C/EBP, CCAAT/enhancer binding protein; CDK, Cyclin dependent kinase; CdX2, Caudal-related homeobox 2; CHOP, CAAT/enhancer binding protein homologous protein; COX-2, Cyclooxygenase-2; CYP7A1, Cholesterol 7alpha-hydroxylase; d, Boswellia; DDIT3, DNA damage inducible transcript 3; DNMT, DNA methyl-transferase; DR, Death receptor; EGR1, Early growth response 1; ERα, Estrogen receptor alpha; FXR, Farnesoid X receptor; G-CSF, Granulocyte colony-stimulating factor; HO-1, Heme oxygenase-1; Hsp90, Heat shock protein 90; IGF1, Insulin-like growth factor 1; JNK, c-Jun N-terminal kinase; IL, Interleukin; IRF3, Interferon-regulatory factor 3; MAPK, Mitogen-activated protein kinase; MCL-1, myeloid leukemia cell differentiation protein; MDR, Multidrug resistance; MMP, Matrix metalloproteinases; MRP, Multidrug resistance protein; mTOR, Mechanistic target of rapamycin; NF-κB, Nuclear factor kappa B; Nrf-2, The nuclear factor erythroid 2 (NFE2)-related factor 2; PARP, Poly ADP ribose polymerase; PI3K, Phosphatidylinositol-4,5-bisphosphate 3-kinase; PPAR, Peroxisome proliferator activated receptor; pRb, phosphorylated Retinoblastoma; PS, Phosphatidylserine; PSA, Prostate-specific antigen; RANKL, Receptor activator of nuclear factor kappa-B ligand; ROS, Reactive oxygen species; SAMD14, Sterile Alpha Motif Domain Containing 14; SHP-1, Src homology region 2 domain-containing phosphatase-1; SMPD3, Sphingomyelin phosphodiesterase 3; STAT, Signal transducer and activator of transcription 6; STAT3, Signal transducer and activator of transcription 3; TGF, Transforming growth factor; TLR-3, Toll-like receptor-3; TLR-4, Toll-like receptor-4; VEGF, Vascular endothelial growth factor; VEGFR2, Vascular endothelial growth factor receptor 2; XIAP, X-linked inhibitor of apoptosis protein*.

## *In vitro* studies with *commiphora* and *boswellia* and their role in different chronic diseases

Numerous *in vitro* studies have indicated the efficiency of guggul against diverse chronic diseases including cancer (Shishodia et al., 2007, 2008; Singh S. V. et al., 2007; Shah et al., 2012; Roy et al., 2016). GS induced apoptosis in cancer cells via inhibition of NF-κB, activation of JNK and downregulation of Akt and anti-apoptotic proteins (Shishodia and Aggarwal, 2004; Shishodia et al., 2007). Treatment with GS led to the inhibition of DNA synthesis and proliferation of leukemia cells via downregulation of cyclin D1, cdc2, and upregulation of p21 and p27 (Samudio et al., 2005; Shishodia et al., 2007). In addition, *B. serrata* gum resin displayed cytostatic and apoptosis-inducing effect against leukemia and brain tumor cells (Hostanska et al., 2002). Further, GS induced cell death in prostate cancer cells by reactive oxygen intermediate (ROI)-dependent activation of JNK, p38 MAPK and also activation of ERK1/2 (Singh S. V. et al., 2007; Xiao and Singh, 2008). Additionally, AKBA inhibited the proliferation and induced apoptosis in colon cancer cells through p21 and caspase-8 dependent pathway (Liu et al., 2002b, 2006). Besides, it also modulated the expression of let-7, miR-200 families and their downstream targets in colon cancer cells (Takahashi et al., 2012).

Apart from cancer, the effect of guggul has been well proven against different inflammatory diseases such as rheumatoid arthritis, IBD, and various other diseases such as obesity, otitis media, uveitis etc. For example, treatment with GS downregulated RANKL induced osteoclastogenesis and blocked IL-1beta mediated production of chemokines and epithelial neutrophil activating peptide-78 (ENA-78), MMP-1,-3 via suppression of NF-κB, nuclear p50, and p65 subunit and IκBα degradation in rheumatoid arthritis (Ichikawa and Aggarwal, 2006; Kinne et al., 2007; Lee et al., 2008; Ammon, 2016). A study conducted by Cheon and group on IBD showed that GS inhibited IL-1beta- or lipopolysaccharide (LPS)- induced ICAM-1 expression, NF-κB transcription activity and IκB phosphorylation/degradation in human Caco-2 cells and rat non-transformed IEC-18 cells (Cheon et al., 2006). Further, treatment with GS alone showed increase in apoptosis and lipolysis and its combination with genistein resulted in increased cleavage of procaspase-3, PARP, expression of Bax, release of cyt-c and prevented lipid accumulation in maturing adipocytes resulting in inhibition of adipogenesis (Yang et al., 2007, 2008). GS exerted its effect against otitis media, the foremost cause of hearing impairment in children by inhibiting LPS-induced upregulation of TNF-α expression, COX-2 production and IκBα degradation (Ovesen and Ledet, 1992; Barrett et al., 2003; Song et al., 2010). In case of uveitis, treatment with GS inhibited LPS-induced expression of inflammatory proteins in human primary non-pigment ciliary epithelial cells (Kalariya et al., 2010).

As guggul is an FXR antagonist, it is used extensively as a cholesterol-lowering agent (Rizzo et al., 2006; Shah et al., 2012). GS eliminated the effect of chenodeoxycholic acid (CDCA), an FXR agonist on the expression of Cdx2 and MUC2 and thus prevented bile acid induced gastric intestinal metaplasia and carcinogenesis (Xu et al., 2010). Oswley and Chiang reported that GS antagonizes FXR induction of BSEP but activates pregnane X receptor to inhibit CYP7A1 gene (Owsley and Chiang, 2003). In addition, *Commiphora* and *Boswellia* showed potent cardioprotective as well as thyroid-stimulatory effects (Singh et al., 1982; Deng, 2007). For instance, GS inhibited DOX induced cytotoxicity, reduced apoptosis, and intracellular ROS and formation of MDA in DOX-treated H9C2 cells (Wang et al., 2012). In addition, triterpenes and prenylaromadendrane-type diterpenes from the gum resin of *B. carterii* was shown to exert hepatoprotective effect against d-galactosamine-induced liver cell damage (Wang et al., 2013, 2016).

## *In vivo* studies with *commiphora* and *boswellia* and their role in different chronic diseases

Promising after effects of *Commiphora* and *Boswellia* against various chronic diseases in the *in vitro* setting has led to a handful of *in vivo* studies where the efficacy of guggul was evaluated in different experimental models of diverse chronic diseases such as cancer, inflammatory, cardiovascular, and metabolic diseases, atherosclerosis, asthma etc. (Table 2; Figure 4). Recently, several studies have reported the anti-tumor efficacy of guggul in different cancers such as cancers of breast, esophagus, head, and neck, pancreas, prostate etc. (An et al., 2009). For instance, AKBA was found to prevent intestinal tumorigenesis and exert chemopreventive effect via inhibition of wnt/β-catenin and NF-κB/COX-2 signaling pathways (Liu et al., 2013; Wang R. et al., 2014). Another study showed AKBA to function via modulation of let-7 and miR-200 downstream genes in colorectal (CRC) tumors (Takahashi et al., 2012). In case of breast cancer, treatment with GS increased the chemosensitivity of MCF-7/DOX cells to doxorubicin *in vivo* through inhibition of Bcl-2 and Pgp (Xu et al., 2014b). In addition, GS suppressed esophageal tumor cell viability via inhibition of FXR and prevented the growth of esophageal cancer cells significantly in combination with amiloride *in vivo* (Guan et al., 2013, 2014). Furthermore, in case of glioma, cyano enone of methyl boswellates (CEMB), and 3-α-propionyloxy-β-boswellic acid (POBA) significantly inhibited the tumor growth in murine models (Ravanan et al., 2011; Qurishi et al., 2013). Again, topical application of Boswellin (BE); *B. serrata* gum resin exudate inhibited skin inflammation, epidermal proliferation, and tumor promotion induced by 12-O-tetradecanoylphorbol-13-acetate (TPA) in 7,12-dimethylbenz[a]anthracene (DMBA)-initiated mice. Additionally, treatment with guggulipid was shown to cause reduced growth of HNSCC cells *in vivo* (Leeman-Neill et al., 2009). Besides, GS enhanced the antitumor efficacy of gemcitabine in pancreatic cancer via modulation of Akt, NF-κB, and apoptosis-related proteins (Ahn et al., 2012). In case of prostate cancer as well, guggul has been found to be highly effective *in vivo* (Syrovets et al., 2005b; Büchele et al., 2006; Pang et al., 2009; Pathania et al., 2015). Oral administration of guggulsterone prevented *in vivo* angiogenesis of prostate cancer cells through suppression of VEGF-VEGF-R2-Akt signaling (Xiao and Singh, 2008).

**Table 2:** *In vivo* biological activities of guggul (*Commiphora* and *Boswellia)* against various chronic diseases.

**Disease**	**Mechanism of action**	**References**
Arthritis	↓Leucocytes	Sharma et al., 1989^d^
	–	Dhaneshwar et al., 2013^d^
	↓IL-1β; ↓TLR4	Wang Q. et al., 2014^d^
Asthma	↓pSTAT6; ↓GATA3	Liu et al., 2015^d^
	↓pSTAT6; ↓GATA3	Zhou et al., 2015^b^
Atherosclerosis	↓NF-κB	Cuaz-Pérolin et al., 2008^d^
**CANCER**		
Breast Cancer	↓PCNA; ↓Ki67	Xu et al., 2014b^c^
Colon Cancer	↑Caspase−3,−8; ↓cIAP-1,-2; ↓Bcl-2	An et al., 2009^c^
	↓Wnt/β-catenin; ↓NF-κB/COX-2	Liu et al., 2013^d^
	↓Wnt/β-catenin; ↓NF-κB/COX-2	Wang R. et al., 2014^d^
	↑let-7 and miR-200 families	Takahashi et al., 2012^d^
Ehrlich tumor	↓VEGF; ↑caspase-3; ↑Bax	Agrawal et al., 2011^d^
	↓NF- κB; ↓PARP	Qurishi et al., 2012^d^
Esophageal Cancer	↓NHE-1	Guan et al., 2014^c^
	↓FXR	Guan et al., 2013^c^
Glioma	↓NO; ↑Caspase–3,–8	Ravanan et al., 2011^d^
	↓ AOM-induced ACF	Huang et al., 2000^d^
	↓NF-κB; ↓PARP	Qurishi et al., 2013^d^
Head and Neck	↓STAT-3; ↓HIF-1α	Leeman-Neill et al., 2009^c^
Pancreatic cancer	↓NF-κB; ↓Akt	Ahn et al., 2012^c^
	↓NF-κB	Park et al., 2011a^d^
Prostate cancer	↓VEGF-VEGF-R2-Akt signaling	Xiao and Singh, 2008^c^
	↓NF-κB	Syrovets et al., 2005b^d^
	↑Caspase-3	Büchele et al., 2006^d^
	↓VEGFR-2	Pang et al., 2009^d^
	↓MMP; ↓PARP-1	Pathania et al., 2015^d^
Colitis	↓NF-κB	Kim et al., 2010^c^
	↓ICAM-1; ↓NF-κB	Cheon et al., 2006^c^
Dementia	↓AChE activity; ↓MDA; ↑GSH	Saxena et al., 2007^c^
Depression	↑BDNF signaling; ↑Hippocampal neurogenesis	Liu et al., 2017^c^
Diabetes	↓PPARγ	Sharma et al., 2009^c^
Gastritis	↓NF-κB	Kim et al., 2013^c^
Gastric injury	↑Nrf2; ↑HO-1	Zhang et al., 2016^d^
Gastric ulcer	↑Prostaglandin; ↓leukotrienes	Singh et al., 2008^d^
Hepatic injury	↓NF-κB; ↓p65; ↓p-JNK; ↓TLR-3,-4; ↓MyD88	Chen et al., 2016^d^
Hyperlipidemia	↓Oxidative modification of LDL	Wang et al., 2004^c^
	–	Satyavati et al., 1969^c^
	↑Plasma insulin level; ↓LDL; ↓VLDL	Sharma et al., 2009^c^
Inflammatory bowel diseases	↓NF-κB	Kang et al., 2013^c^
	↓IL–2,−4; ↓IFN-γ	Mencarelli et al., 2009^c^
Ischemia reperfusion	↑Nrf2; ↑HO-1	Ding et al., 2014^d^
	↑Nrf2; ↑HO-1	Ding et al., 2015^d^
Memory impairment	↑CREB-BDNF signaling	Chen et al., 2016^c^
Myocardial ischemia	↓Oxidative degradation of lipids; ↓ROS	Chander et al., 2003^c^
	↓Lipid peroxides; ↓XO; ↑SOD	Kaul and Kapoor, 1989^c^
Pancreatitis	↓NF-κB; ↓IL-6; ↓Chemokine-1,-10	Kim et al., 2017 ^c^
Thyroid dysfunction	↑Iodine uptake	Tripathi et al., 1975^c^, 1984^c^
	↑Iodine uptake	Panda and Kar, 2005
Uveitis	↓NF-κB; ↓MMP-2; ↓iNOS; ↓COX-2	Kalariya et al., 2010^c^

*ACF, Aberrant crypt foci; AChE, Acetylcholinesterase; AOM, azoxymethane; BCRP, Breast cancer resistance protein; BDNF, Brain-derived neurotrophic factor; c, Commiphora; cIAP, The cellular inhibitor of apoptosis; COX-2, Cyclooxygenase-2; CREB-BDNF, cAMP-response element binding protein-BDNF d, Boswellia; FXR, Farnesoid X receptor; GSH, Glutathione; HIF-1a, Hypoxia-inducible factor 1-alpha; HO-1, Heme oxygenase-1; ICAM, Intracellular adhesion molecules; IFN, Interferon; IκB, Nuclear factor of kappa light polypeptide gene enhancer in B-cells inhibitor IL, Interleukin; IL-1β, Interleukin 1 beta; iNOS, Inducible nitric oxide synthase; LDL, Low density lipoprotein; MDA, Malondialdehyde; MDR, Multidrug resistance; MMP, Matrix metalloproteinases; MyD88, Myeloid differentiation primary response 88; NF-κB, Nuclear factor kappa B; NHE-1, Na+/H+exchanger-1; NO, Nitric oxide; Nrf-2, The nuclear factor erythroid 2 (NFE2)-related factor 2; p-JNK, Phosphorylated Jun N-terminal kinases; P-gp, P-glycoprotein; PARP, Poly ADP ribose polymerase; PCNA, Proliferating cell nuclear antigen; PPAR, Peroxisome proliferator activated receptor; pSTAT6, phosphorylated Signal transducer and activator of transcription 6; ROS, Reactive oxygen species; SOD, Superoxide Dismutase; STAT, Signal transducer and activator of transcription; TLR, Toll-like receptor; VEGF, Vascular endothelial growth factor; VEGFR2, Vascular endothelial growth factor receptor 2; XO, Xanthine oxidase*.

Apart from cancer, the efficacy of guggul was well proven in different inflammatory diseases such as arthritis, colitis, gastritis, IBD, pancreatitis, uveitis etc. (Sharma et al., 1989; Cheon et al., 2006; Xiao and Singh, 2008; Mencarelli et al., 2009; Kalariya et al., 2010; Kim et al., 2010, 2013; Dhaneshwar et al., 2013; Kang et al., 2013; Wang R. et al., 2014). In case of rheumatoid arthritis, treatment with guggul decreased the thickness of joint swelling, reduced the infiltration of leucocytes into the pleural cavity, suppressed the pro-inflammatory cytokines and increased beta-glucuronidase activity *in vivo* (Sharma and Sharma, 1977; Reddy and Dhar, 1987; Sharma et al., 1989; Fan et al., 2005). In addition, guggul reduced the severity of IBD via inhibition of LPS- or IL-1beta-induced ICAM-1 gene expression and NF-κB activity (Krieglstein et al., 2001; Cheon et al., 2006; Mencarelli et al., 2009; Kim et al., 2010). Furthermore, administration of GS resulted in mitigation of histological damage, suppressed serum lipase levels, inhibition of infiltrations of neutrophils, and macrophages and decreased cytokine production in pancreatitis (Kim et al., 2015). Moreover, GS inhibited the expression of endotoxin-induced uveitis (EIU)-associated inflammatory markers such as MMP-2, NO, and prostaglandin E_2_ (PGE_2_) (Kalariya et al., 2010).

Guggul exhibited profound cardioprotective effects as well *in vivo* (Chander et al., 2003). It decreased the lipid peroxide, creatine phosphokinase, phospholipase, xanthine oxidase activities, and total cholesterol level in the serum; increased superoxide dismutase (SOD), myocardial antioxidants, glutathione peroxidase (GSHPx), catalase (CAT); reduced glutathione (GSH), creatine-phosphokinase-MB (CK-MB), and lactate dehydrogenase (LDH) as well as reversed the cardiac damage induced by isoproterenol (Kaul and Kapoor, 1989; Batra et al., 2000; Ojha et al., 2011). The hypolipidemic effect of guggul has also been well studied in different animal models (Khanna et al., 1969; Dixit et al., 1980; Baldwa et al., 1981; Lata et al., 1991). Guggul diminished hyperlipidemia via inhibition of FXR activation. In high-fat-diet-fed mice, treatment with GS improved blood glucose in fasting condition, plasma insulin level, glucose tolerance, level of harmful lipids, phosphoenol pyruvate carboxykinase, glucose-6-phosphatase, and other proteins like glucose transporter-4, PPARc, and TNF-α (Satyavati et al., 1969; Singh et al., 1990; Urizar et al., 2002; Cui et al., 2003; Sharma et al., 2009; Tripathi, 2009). Further, *C. opobalsamum, C. mukul, B. serrata*, and *B. ovalifoliolata* species mitigated hepatic damage and displayed protective effect against lipid peroxidation and deviated serum enzymatic variables (Al-Howiriny et al., 2004; Y et al., 2006; Mahesh et al., 2014). In addition, GS reversed neuronal damage and memory deficits in mice by increasing glutathione level in the brains, antiacetylcholine esterase, and antioxidant activities (Saxena et al., 2007). Apart from these, administration of GS was found to increase thyroid function by enhancing iodine uptake, improved the activities of thyroid peroxidase, and protease and ameliorated hypothyroidism through its ability to increase thyroid hormone *in vivo* (Tripathi et al., 1975, 1984; Panda and Kar, 2005).

Taken together, these pre-clinical studies provide substantial evidence of the enormous potential of guggul as a multi-targeted agent for the prevention and treatment of different chronic diseases.

## Clinical studies with *commiphora* and *boswellia* and their role in different chronic diseases

Several clinical trials have been conducted to evaluate the effect of “guggul” from *Commiphora* and *Boswellia* on various chronic disorders. Human studies on guggul has been found to be effective against different diseases such as asthma, breast fibroadenoma, chronic kidney disease, colitis, Crohn's disease, fascioliasis, hepatitis C, hypercholesterolaemia, hyperlipidemia, metabolic syndrome, nodulocystic acne, arthritis, schistosomiasis, stress urinary incontinence etc. (Table 3; Figure 4).

**Table 3:** Clinical trials of guggul (*Commiphora* and *Boswellia*) against various chronic diseases.

**Disease**	**Dose**	**Pts (#)**	**Clinical outcome**	**References**
Healthy volunteer	1 g^b^	10	Diminished efficacy	Dalvi et al., 1994
	125 mg, 2 capsules^d^	20	Increased pain threshold & tolerance force, well -tolerated	Prabhavathi et al., 2014
	140 mg^e^	47	Effective	Chilelli et al., 2016
	2 × 250 mg^d^	12	High and quick absorption	Riva et al., 2016
Asthma	900 mg/*d*; 6 wk^d^	40	Improved disease condition	Gupta et al., 1998
	500 mg/*d*^d^	32	Effective	Ferrara et al., 2015
Breast fibroadenomas	–^e^	64	Reduction in fibroadenoma mass	Pasta et al., 2016
CKD	516 mg^e^	16	Safe and tolerable	Moreillon et al., 2013
	–^b^	60	Effective	Shelmadine et al., 2017
Colitis	900 mg/*d*; 6 wk^d^	30	Safe and effective	Gupta et al., 2001
	1050 mg/*d*; 6 wk^d^	–	Effective	Gupta et al., 1997
Crohn's disease	–^d^	102	Safe and effective	Gerhardt et al., 2001
	2,400 mg/*d*; 52 wk^d^	108	Well-tolerated	Holtmeier et al., 2011
Fascioliasis	12 mg/kg/*d*; 6 *d*^f^	7	Safe, well-tolerated and effective	Massoud et al., 2001
	600 mg/*d*; 6 *d*^f^	1019	Safe and effective	Abo-Madyan et al., 2004b
Hepatitis C	–^a^	15	Effective	Scholtes et al., 2012
HCL	1,500 mg/*d*; 12 wk^b^	205	Effective	Nityanand et al., 1989
	100 mg/*d*; 24 wk^f^	61	Mild side effects	Singh et al., 1994
	1,000, 2,000 mg/*d*; 3 d^b^103	–	Well-tolerated, caused dermatologic hypersensitivity	Szapary et al., 2003
	2,160 mg/*d*; 12 wk^b^	43	Clinical magnitude is obscure	Nohr et al., 2009
HLD	–^f^	–	–	Verma and Bordia, 1988
	75 mg/*d*; 8 wk^a^	–	Safe and effective	Beg et al., 1996
	2 g/*d*; 8 wk^b^	59	Effective	Vyas et al., 2015
Metabolic syndrome	2 pills/*d*; 4 mo^b^	78	Effective	Patti et al., 2015
Nodulocystic acne	50 mg/*d*; 3 mo^b^	20	Reduced inflammatory lesions	Thappa and Dogra, 1994
Osteoarthritis	2 capsules, every 8 h; 3 m-15 d wash-out-3 m^e^	42	–	Kulkarni et al., 1991
	500 mg^b^	30	Safe and effective	Singh et al., 2003
	999 mg/*d*; 8 wk^d^	30	Well-tolerated	Kimmatkar et al., 2003
	100 or 250 mg/*d*; 90 *d*^d^	75	Safe and effective	Sengupta et al., 2008
	1000 mg/*d*^e^	30	Safe and well-tolerated	Kizhakkedath, 2013
	6 capsules/*d*; 24 wk^e^	440	Effective, improved knee function	Chopra et al., 2013
Polyarthritis	3600 mg/*d*^d^	78	No measurable efficacy	Sander et al., 1998
RT-related edema	4200 mg/*d*^d^	44	Effective, reduced cerebral edema	Kirste et al., 2011
Schistosomiasis	10 mg/kg/*d*; 3*d*^f^	204	Well-tolerated	Sheir et al., 2001
	600 mg/*d*; 6 *d*^f^	1019	Safe and effective	Abo-Madyan et al., 2004a
Skin damage in	Cream, twice/*d*^d^	114	Well-tolerated	Togni et al., 2015
MCA				
SUI	4 g/*d*, 8 wk^e^	30	Effective	Arkalgud Rangaswamy et al., 2014

*CKD, Chronic kidney disease; d, Day; HCL, Hypercholesterolemia; HLD, Hyperlipidemia; MCA, Mammary carcinoma; mo, Month; wk, Week; RT, Radiotherapy; SUI, Stress urinary incontinence; a, Gugglusterone; b, Guggul; c, Formulation of guggul; d, Boswellia; e, Formulation of Boswellia; f, Commiphora*.

### Arthritis

Arthritis is mainly caused due to inflammation of joints, the tissues surrounding the joints and other connective tissues. Osteoarthritis is the most common form of arthritis which affects a wide range of people across all the places. As guggul has been reported to exhibit high affectivity against arthritis pre-clinically; hence, its effect was evaluated in the clinical setting as well. In one such study, 30 patients with arthritis were treated with gum guggul for 1 month which resulted in remarkable improvement in the total scores of Western Ontario and MacMaster Osteoarthritis Index and condition of the patients (Singh et al., 2003). Another study was conducted by Kimmatkar et al., to check the safety, tolerability, and efficacy of *B. serrata* extract in 30 patients with knee osteoarthritis. The patients receiving drug treatment reported a decrease in knee pain and swelling of the knee joint as well as increased knee flexion and walking distance (Kimmatkar et al., 2003).

### Asthma

Asthma is a chronic multifactorial inflammatory disease of the respiratory tract and is one of the major health concern. Notably, *Boswellia* has been found to be effective in the treatment of this disease. In a clinical study, 40 patients having 23 males, and 17 females in the age range of 18–75 years, suffering from bronchial asthma were treated with 300 mg of gum resin thrice daily for a period of 6 weeks. This led to improved prognosis in around 70% of the patients as various signs and symptoms of bronchial asthma like rhonchi, dyspnoea, and attacks disappeared upon treatment (Gupta et al., 1998).

### Breast fibroadenomas

Breast fibroadenoma accounts for the majority of breast lumps in young women. *Boswellia* was found to exert beneficial effect against breast fibroadenomas as evinced by a study conducted by Pasta and group. They showed that treatment with the combination of *Boswellia*, betaine, and myo-inositol resulted in decreased fibroadenoma dimension in young women without exerting any toxic effects. The combination also resulted in reduced fibroadenoma volume in 38.8% of the patients in the experimental group, whereas the same was observed only in 17.85% patients in the placebo group (Pasta et al., 2016).

### Cardiovascular diseases (CVDS)

CVDs, a group of diseases which involves the heart and the blood vessels is one of the most common causes of death across the globe. Notably, guggul presents a potent remedy for cardiovascular diseases. For example, Singh and group conducted a study to evaluate the cardioprotective benefits of guggul by enrolling 200 patients suffering from ischemic heart disease. The patients were treated with the combination of gum guggul and *Inula racemosa* for 6 months which resulted in the reduced levels of total cholesterol, triglyceride, and total blood lipids in the patients. It also restored the normal electrocardiogram (ECG) in 26% of the patients, showed improvement of ECG in 59% of the patients and lessened the chest pain in 25% of the patients (Singh et al., 1993).

### Chronic kidney disease

Chronic kidney disease (CKD) is a progressive disease where occurs due to enhanced inflammation and oxidative stress leading to reduced kidney function. Studies have indicated *B. serrata* in combination with *Curcuma longa* as an effective regimen to obtain reduced inflammation in patients with CKD which functioned via modulation of prostaglandin E_2_ (PGE_2_) (Shelmadine et al., 2017). Moreover, this regimen was found to be safe, well tolerated which also enhanced the levels of inflammatory cytokines in CKD patients (Moreillon et al., 2013).

### Diabetes mellitus

A large population of the world is affected by diabetes mellitus or type 2 diabetes. Several preclinical studies have shown that the gum resin of *commiphora* and *boswellia* are highly effective against this disease. In a clinical study conducted by Ahangarpour et al., it was observed that the treatment of patients with diabetes mellitus with *B. serrata* gum resin (900 mg daily for 6 weeks orally) resulted in decreased risk factors associated with this disease. Further, the treatment also helped in maintaining fructosamine levels, hepatic enzyme activities, and to bring lipid profiles close to normal levels in the patients (Ahangarpour et al., 2014).

### Eczema and psoriasis

Eczema, also known as dermatitis and psoriasis are caused mainly due to inflammation of the skin. *Boswellia* has been found to exert effectiveness against eczema and psoriasis. A group of scientists revealed that *Boswellia*-based cream lessens the use of topical corticosteroids and can diminish the grade of erythema and the skin superficial symptoms (Togni et al., 2015). Further, in a double blind study, the efficacy of a novel formulation of BA (Bosexil®) containing *B. serrata* resin extract and lecithin was evaluated against both psoriasis and eczema. Improvement in psoriasis, scales (70% of cases), and erythema (50% of cases) was observed with Bosexil® compared to placebo. In addition, when eczema patients were administrated with Bosexil® formulation, it showed improvement in both erythema (60% of cases) and itch (60% of cases) of the patients without any case of waning (Togni et al., 2014).

### Fascioliasis

Fascioliasis is a parasitic worm infection caused by the common liver fluke *Fasciola hepatica* and *Fasciola gigantica*. The formulation of myrhh, the gum resisn of *Commiphora molmol* was reported to be safe, well tolerated, and effective for the management of this disease. The formulated drug comprised of 8 parts of resin and 3.5 parts of volatile oils, all extracted from myrrh. They observed that 7 patients who were passing fasciola eggs in their stools displayed distinct improvement of the general condition, drop in the egg count, and improvement of all signs and symptoms with no adverse side effects after treatment with the drug (Massoud et al., 2001).

### Gingivitis

Gingivitis, the inflammation of gingiva is a very common form of gum disease. Frankincense extract has been found to exhibit efficacy against gingivitis. A double blinded randomized placebo controlled trial was conducted among 75 female patients aged between 15 and 18 years with moderate plaque-induced gingivitis. Six groups were randomly formed based on the administration of 0.1 g of frankincense extract, 0.2 g of its powder, placebo, and whether the patients have undergone scaling and root planning (SRP) or not. Gingival index, plaque index, bleeding index, and probing pocket depth were measured on the 0, 7th, and 14th days of the study. Detailed analysis of the data revealed that SRP along with the application of frankincense extract or powder might cause significant decrease in inflammatory indices in comparison to the groups without drug therapy and SRP (Khosravi Samani et al., 2011).

### Inflammatory bowel disease

Different clinical studies with guggul have shown its efficacy against IBDs which include colitis and Crohn's disease. For instance, the gum resin of *B. serrata* was found to be effective in the treatment of chronic colitis with minimal side effects in a clinical study conducted by Gupta et al. In this study, the patients with chronic colitis were treated with gum resin from *B. serrata* at a dose of 900 mg daily divided in three doses for 6 weeks. The treatment resulted in the improvement of stool properties, hemoglobin, serum iron, calcium, phosphorus, proteins, total leukocytes, and eosinophils in the patients (Gupta et al., 2001). Further, a double-blind, placebo-controlled, randomized, parallel study on 82 patients with Crohn's disease was conducted where patients were given a new *B. serrata* extract; Boswelan. In this trial, remission was observed in 59.9% of the actively treated patients. Additionally, this study also confirmed better tolerability of Boswelan in long-term treatment of Crohn's disease (Holtmeier et al., 2011). Furthermore, leukotrienes play an important role in inflammation of the colon in ulcerative colitis. Sallai guggul gum resin is known to be specific, non-redox, and non-competitive inhibitors of 5-LOX, a crucial enzyme of leukotriene biosynthesis. Patients with grade II and III ulcerative colitis were treated with *B. serrata* gum resin at a dose of 350 mg thrice daily for 6 weeks. Stool properties, histopathology, and scan microscopy of rectal biopsies, blood parameters including hemoglobin, serum iron, calcium, phosphorus, proteins, total leukocytes, and eosinophils showed slightly better improvement in *Boswellia* treated patients (Gupta et al., 1997).

### Nodulocystic acne

Guggulipid is considered to be very effective in topical and oral complementary as well as an alternative medicine (CAM) for the treatment of acne (Magin et al., 2006). In a clinical study conducted by Thappa and Dogra, patients with nodulocystic acne were given guggulipid equivalent to 25 mg GS for 3 months, which resulted in progressive reduction in lesions in majority of patients. However, patients with oily faces displayed better response to guggulipid (Thappa and Dogra, 1994).

Thus, these clinical studies well evince the potential effect of *Commiphora* and *Boswellia* on different chronic diseases. However, more studies are inevitable to establish them as cutting edge strategy for the treatment of diverse human diseases.

## Conclusion

Since ancient times, *Commiphora* and *Boswellia* are considered as important traditional medicinal plants which are used for the treatment of various ailments. Guggul isolated from *Commiphora* and *Boswellia* have immense therapeutic potential against several diseases and it has been well established by numerous *in vitro, in vivo*, and clinical studies. Guggul was used traditionally for the treatment of inflammation and hyperlipidemia, but with the extensive studies on guggul and associated molecular mechanisms unveiled newer insights of its use for the treatment of various other chronic diseases as well. Gum resin guggul possesses multiple pharmacological activities especially hypolipidemic, antiobesity, anti-inflammatory, anti-tumor effects, cardioprotective, neuroprotective, hepatoprotective, thyroid stimulatory effects etc. It effectively regulates different transcription factors, enzymes, cytokines, and anti-apoptotic proteins which are involved in inflammation, carcinogenesis, and other chronic diseases. Further, *Commiphora* in combination with other ayurvedic herbs is commercially available and marketed for the treatment and cure of arthritis, obesity and associated side effects of the disease. Many patents are also filed and approved to use guggul as a constituent of polyherbal formulations and cosmetics. Therefore, taking the medicinal importance and commercial use of guggul into consideration, it can be advocated to possess substantial therapeutic potential against diverse chronic disorders. However, more *in vitro, in vivo*, and well-designed clinical studies are required to validate the clinical usefulness of guggul and to obtain a potent herbal derived drug with enhanced efficacy, minimal side effects and strong disease combating properties.

## Author contributions

BA and AK contributed to study design and writing of the manuscript. KB and DB carried out literature survey, writing and artwork. CH, BS, and NR contributed to the making of the tables and artwork. SG and GP performed proofreading of the manuscript.

### Conflict of interest statement

The authors declare that the research was conducted in the absence of any commercial or financial relationships that could be construed as a potential conflict of interest.
